# Illiteracy and diabetes: educational program for people with type 2 diabetes in the public health system

**DOI:** 10.1186/1758-5996-7-S1-A177

**Published:** 2015-11-11

**Authors:** Sheila Mara Silva de Oliveira, Débora Bohnen Guimarães, Janice Sepúlveda Reis

**Affiliations:** 1 Instituto de Ensino d Pesquisa Santa Casa – BH, Belo Horizonte, Brazil

## Background

Diabetes (DM) is a public health problem in Brazil and illiteracy is an obstacle in controlling the disease, with this population often not answered properly in Public Health System (PHS).

## Objective

To develop and evaluate educational material to people with type 2 diabetes with limited health literacy or illiteracy, attended by PHS.

## Materials and methods

Educational material was prepared with pictures of food and portion sizes, corresponding to eating plan and based on calories and food groups and list of drugs identified by different colors. Health literacy was measured with the brief-form Test of Functional Health Literacy (B-TOFHLA) in 53 patients with type 2 DM in a single Public Health Center in Belo Horizonte-MG. Educational program was conducted in four weekly meetings, in groups with 10 people, directed to use of drugs and nutrition, advising how to identify drugs by different colors, guiding meals with an individualized eating plan and a list of food adapted to this reality. The meetings were based on educational material. Clinical evaluation and biochemical tests were analyzed before and three months after the last meeting.

## Results

Forty patients were included in the study, classified as inadequate or marginal health literacy. At 3 months, there were improvements in fasting glucose (p 0.005), A1C (p 0.018), total cholesterol (p 0.006) and LDL cholesterol (p 0.014) and reduction in systolic (p 0.005) and diastolic (p 0.018) blood pressure. There were no changes in weight, BMI and abdominal girth or in dose of medication and level of physical activity throughout the study.

## Conclusion

The clear and simple language of the educational material proved to be feasible in primary care with the changes of behavior in patients with type 2 DM illiterate and functionally illiterate, generating greater control of this condition. Diabetes educators must recognize that inadequate literacy is common and that diabetes care can be even more challenging for this group.

**Figure 1:**
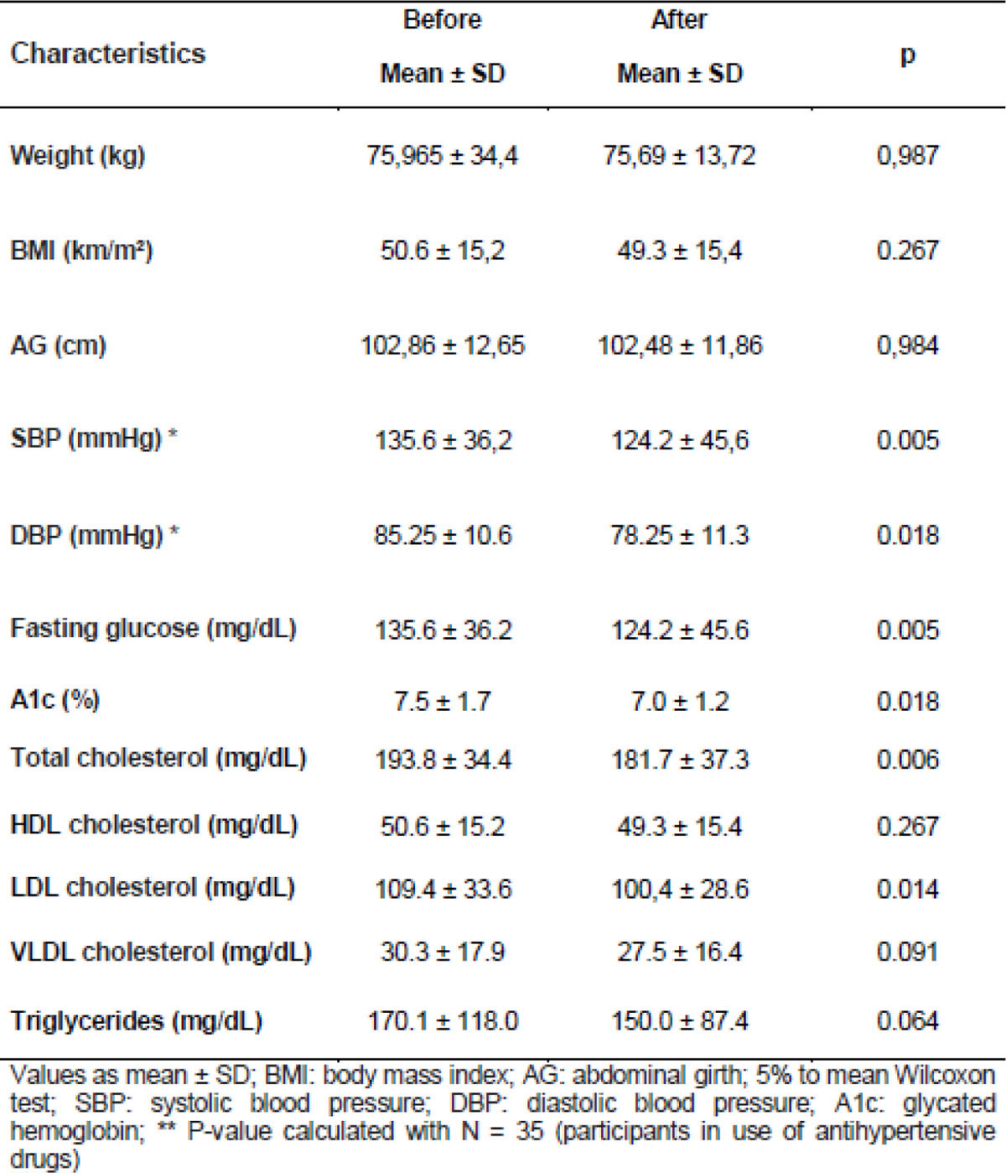
Comparison of the clinical and biochemical variables before and after educational program.

